# The Past, Present, and Future Perspective of Photodynamic Therapy for Age-Related Macular Degeneration

**DOI:** 10.3390/jcm14041240

**Published:** 2025-02-13

**Authors:** Shigeru Honda

**Affiliations:** Department of Ophthalmology and Visual Sciences, Osaka Metropolitan University Graduate School of Medicine, Osaka 545-8585, Japan; shonda@omu.ac.jp; Tel.: +81-6-6645-3867; Fax: +81-6-6634-3873

**Keywords:** photodynamic therapy, age-related macular degeneration, anti-VEGF therapy, combination therapy, polypoidal choroidal vasculopathy

## Abstract

Photodynamic therapy (PDT) has been approved as a therapeutic modality for the management of neovascular age-related macular degeneration (nAMD). PDT can treat macular neovascularization with minimal effects on normal tissue, reducing lesion size and maintaining patients’ visual acuity; consequently, it became the choice of intervention for nAMD around the year 2000. However, the visual outcomes of PDT are inferior to those of anti-vascular endothelial growth factor therapy. Thus, PDT is no longer favored, except for the management of some specific phenotypes of nAMD (e.g., polypoidal choroidal vasculopathy and pachychoroid neovasculopathy) that are good candidates for PDT. Despite these circumstances, PDT remains an effective treatment modality for several retinal and choroidal diseases and should be considered for further use. This review summarizes the past and present positions of PDT in the field of ophthalmology and discusses the future perspectives on PDT.

## 1. Introduction

Photodynamic therapy (PDT) was introduced to the field of ophthalmology in 2000 as a treatment modality for the management of neovascular age-related macular degeneration (nAMD). Prior to its introduction, the treatment modalities for nAMD comprised the coagulation of macular neovascularization (MNV) and normal retinal tissue using a thermal laser; the surgical removal of MNV; and the creation of a circumferential retinal incision, the rotation of the retina, and the displacement of the macula away from MNV. However, these treatment modalities are not used routinely as they are associated with serious complications. PDT has attracted attention in recent years owing to its ability to act only on MNV, thereby minimizing its effects on normal tissues. Thus, it has become the choice of treatment for nAMD since its introduction. Notably, the introduction of anti-vascular endothelial growth factor (VEGF) therapy, which has demonstrated greater efficacy in enhancing the visual outcomes, has led to a decrease in the use of PDT, particularly in non-Asian countries. However, the reuse of PDT, with a particular focus on its ability to reduce the lesions associated with polypoidal choroidal vasculopathy (PCV, a type of nAMD prevalent in Asia [including Japan]) and its therapeutic effect on pachychoroid spectrum diseases (PSD, a group of diseases affecting the posterior pole of the fundus characterized by the presence of dilated large choroidal vessels), has become a hot topic of research in recent years. This review aims to provide an overview of the principles and history of PDT in the field of ophthalmology and the current indications for PDT. In addition, this study also discusses future perspectives on this modality.

## 2. Introduction of PDT to Ophthalmology and Subsequent Changes

### 2.1. PDT for the Treatment of nAMD

**Figure 1:**
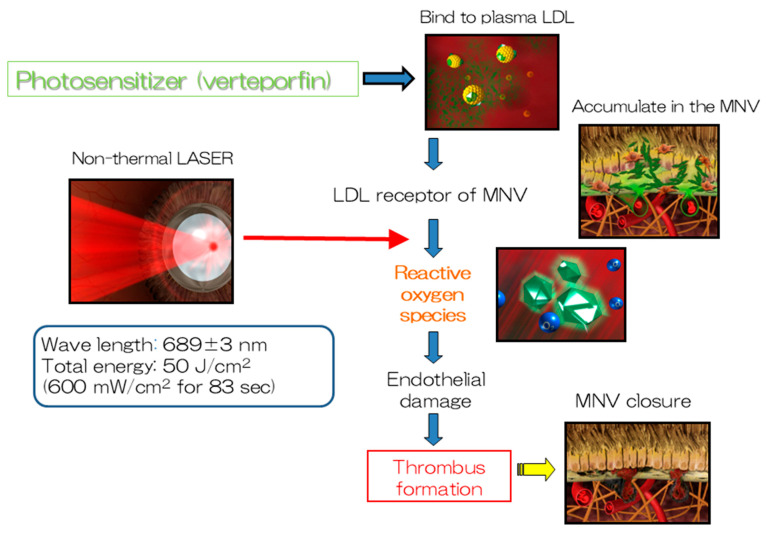
The mechanism of action of PDT. PDT produces reactive oxygen species that facilitate thrombus formation at macular neovascularization by inducing endothelial damage.

The treatment for nAMD involves intravenously injecting verteporfin (Visudyne^®^), the photosensitive substance used in PDT, over a period of 15 min, which binds to low-density lipoprotein (LDL) in the blood. LDL receptors are abundant in the endothelial cells of MNV; consequently, verteporfin is taken up by the endothelium of MNV. The irradiation of the lesion with a non-heat-generating laser is commenced for 90 s after confirming a sufficient uptake of verteporfin to facilitate its activation. The singlet oxygen released by the activated verteporfin promotes thrombus formation by damaging the endothelial cells, thereby occluding the MNV (Figure 1). Patients are monitored after the initial treatment; retreatment can be initiated at intervals of >3 months, if necessary. The Treatment of Age-related Macular Degeneration with Photodynamic Therapy (TAP) Study [1], a randomized, double-blind, prospective study, was the first study to use PDT in the field of ophthalmology. A total of 609 patients with type 2 MNV in nAMD, which is characterized by the presence of mesh-like MNV, were recruited from Europe and the United States and compared with 402 patients in a PDT group and 207 patients in a placebo group. The rate of moderate or severe visual loss (≥15 letters on the ETDRS chart visual acuity test) was significantly lower in the PDT group (39%) compared with that in the placebo group (54%) after 1 year. Furthermore, an improvement of ≥5 letters was observed in 16% and 7% of the patients in the PDT and placebo groups, respectively. The average visual acuity in the PDT and placebo groups was 10.8 letters and 17.6 letters, respectively, demonstrating the significant ability of PDT to preserve visual acuity. In addition, the rate of the complete occlusion of MNV in the PDT group was over twice as high as that in the placebo group (19% vs. 9%, respectively). Moderate or severe visual loss was observed after 2 years in 47% and 62% of the patients in the PDT and placebo groups, respectively. The mean visual acuity in the PDT and placebo groups was 13.4 letters and 19.6 letters, respectively. The patients included in the TAP study received 3.4 treatments and 5.6 treatments on average in the first year and over a period of two years, respectively. A non-occluded extension study involving 320 of 402 patients included in the initial PDT group in the TAP study was conducted after two years. Notably, 193 of these 320 (60%) patients were treated and followed up for an additional three years (total of five years). An average visual acuity loss of 7.5 letters was observed in these patients over the two years of the main TAP study; however, the mean visual acuity loss was only 8.0 letters (an additional loss of 0.5 letters) at the end of the extension study (after five years). Many participants dropped out from the extension study; this may have affected the results. Nevertheless, PDT was considered to have a lesion-suppression effect. The Verteporfin in Photodynamic Therapy (VIP) study [2], a randomized, double-blind, prospective study involving 339 patients with type 1 MNV in nAMD conducted concurrently with the TAP study, compared 225 patients with nAMD who received PDT with 114 patients with nAMD who received placebo treatment. The proportion of patients in the PDT group (54%) with moderate or severe visual impairment after two years was significantly lower than that in the placebo group (67%). Similarly, the proportion of patients with severe visual impairment in the PDT group (30%) was also significantly lower than that in the placebo group (47%). In particular, the proportion of patients with moderate or severe visual impairment after two years was significantly lower in the PDT group than that in the placebo group when the lesion size was ≤4 disc areas or the visual acuity score was ≤65 letters (equivalent to 0.4 decimal visual acuity). The patients received an average of five treatments over 2 years, which was almost identical to that in the TAP study. Multivariate analyses of the TAP and VIP studies revealed that a smaller lesion size was associated with a significantly better visual prognosis. The Japanese Age-Related Macular Degeneration Trial (JAT) study [3], the first clinical trial of PDT conducted in Japan based on the findings of the TAP and VIP studies, was a one-year unobstructed clinical trial involving 64 patients with nAMD. The primary endpoint of visual acuity, which was a mean of 50.8 letters at baseline, improved to 53.8 letters at 12 months, indicating the maintenance of or slight improvement in the visual acuity. The mean lesion size decreased from 3229 mm at baseline to 1260 mm at 12 months. A placebo group was not included in this study; however, the change in visual acuity among the enrolled patients was observed until the commencement of the study. The average visual acuity of 74.9 letters 9–15 months before the commencement of the study decreased to 51.3 letters immediately before the commencement of the study despite variations in the number of cases. Thus, the tendency of the average visual acuity to improve following PDT was the biggest finding of this study, which differs from the findings of the TAP and VIP studies that only reported a decline in the average visual acuity and suggested that the type of nAMD affecting patients in Japan may vary from that affecting those in Europe and the United States. A post-hoc analysis of the JAT study conducted later revealed that this finding can be attributed to PCV, which is abundant among patients with nAMD in Japan, exhibiting an excellent response to PDT [4]. Notably, several facilities have reported favorable therapeutic effects of PDT in patients with PCV.

### 2.2. Emergence of Anti-VEGF Therapy and the Decline in the Popularity of PDT

**Figure 2:**
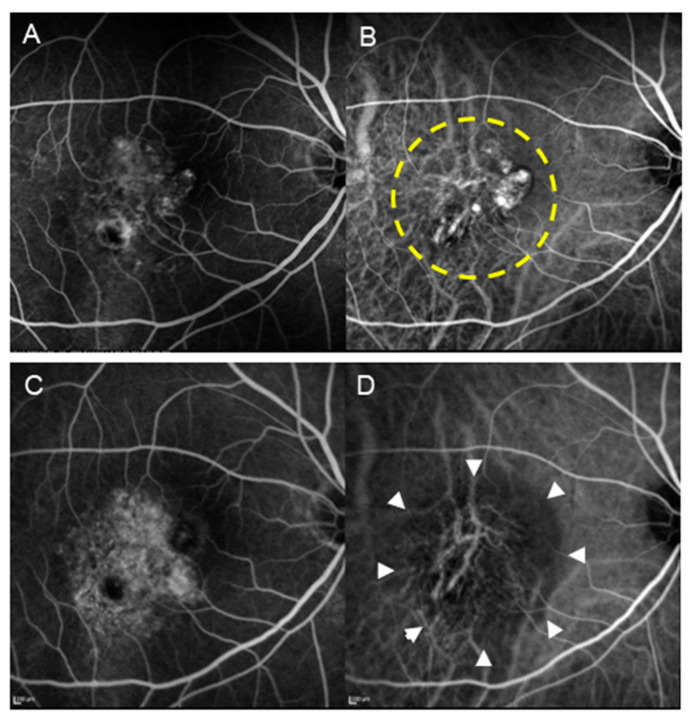
A patient with PCV (70-year-old male) who underwent PDT monotherapy. (**A**) Pre-treatment FA revealed type 1 MNV. (**B**) Pre-treatment IA shows the presence of blanching vascular networks and polypoid lesions. The area enclosed by the dashed line underwent laser irradiation. (**C**) Post-treatment FA revealed increased tissue staining. (**D**) In IA after treatment, regression of polypoid lesions and occlusion of the choriocapillaris (encircled by arrowheads) are seen.

Anti-VEGF therapy was introduced as a treatment modality for nAMD in the 2000s. Three anti-VEGF drugs have been approved for use in Japan since 2008: the anti-VEGF aptamer pegaptanib, the anti-VEGF neutralizing antibody Fab fragment ranibizumab in 2009, and the VEGF receptor-1,2 and Fc chimeric protein aflibercept in 2012. Notably, pegaptanib was later withdrawn from the market. The MARINA study [5] and the ANCHOR study [6], two clinical trials that were conducted to evaluate the efficacy of anti-VEGF therapy in the management of nAMD, revealed that the administration of ranibizumab resulted in a significant improvement in vision among patients with typical nAMD. The post-treatment course of anti-VEGF treatment is superior to that of control and PDT groups; consequently, the use of PDT has been almost completely discontinued in Europe and the United States following the introduction of anti-VEGF treatment. However, the prevalence of PCV, a disease similar to nAMD characterized by the presence of abnormal polypoid blood vessels, is high in Asia. PDT had a significant effect on regressing the polypoid lesions of PCV in several studies conducted mainly in Asia (Figure 2). Moreover, the accompanying exudative changes also exhibited significant improvement [7,8,9,10,11]. Therefore, PDT, an effective treatment modality for PCV, remains a treatment alternative in Asian countries.

**Figure 3:**
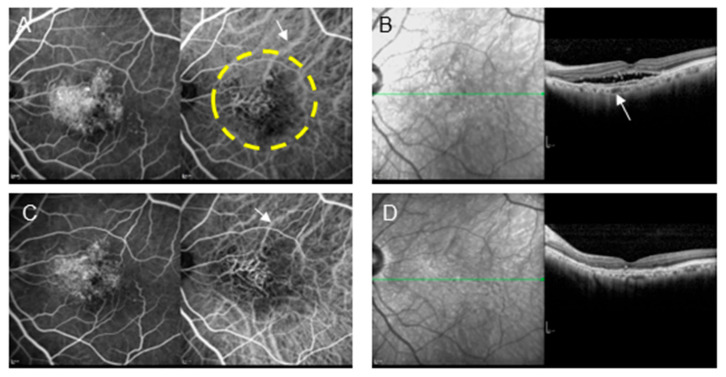
A patient with type 1 MNV (72-year-old male) who refused anti-VEGF therapy owing to a history of brain infarction and underwent PDT monotherapy. (**A**) Pre-treatment FA and IA show MNV and some choroidal vascular dilation (arrow). The area enclosed by the dashed line underwent laser irradiation. (**B**) OCT shows pigment epithelial detachment (PED) and subretinal fluid. The choroidal thickness is relatively thin; however, some large blood vessels are dilated (arrow). (**C**) Post-treatment FA shows tissue staining. In IA, the size of the MNV did not change; however, choroidal vascular contraction (arrow) was observed. In addition, occlusion of the choriocapillaris was observed in the area where the laser was applied. (**D**) Post-treatment OCT shows the disappearance of subretinal fluid and decreased choroidal thickness, which is a critical effect of PDT to reduce disease activity.

The LAPTOP study [12,13,14], conducted in Japan involving patient with PCV only, revealed that the progression of visual acuity following PDT alone was inferior to that observed following treatment with ranibizumab. This may be attributed to treatment with PDT alone being associated with a certain probability (approximately 15% at 12 months in the LAPTOP study) of significant visual impairment being observed following treatment with PDT, only owing to the development of complications such as the atrophy of the choriocapillaris and retinal pigment epithelium cells (Figure 2 and Figure 3) and massive subretinal hemorrhage during the early postoperative period. Thus, PDT alone (excluding reduced PDT, which halves the amount of laser irradiation) is no longer performed routinely. These complications have been attributed to the significant inflammatory reaction and increased VEGF levels observed early after PDT. The incidence of PDT-induced acute exudative maculopathy (PAEM), an adverse condition that develops a few days after undergoing PDT, has been proposed in recent years [15,16,17,18]. PAEM is characterized by an increase in serous retinal detachment and PED owing to an acute fibrinous inflammatory process; this may lead to vision loss during the early stages of treatment. The incidence and time course of PAEM in patients with nAMD, PCV, and central serous chorioretinopathy (CSC) were evaluated in a recent study. The incidence of PAEM among patients with PCV and CSC was significantly lower than that among those with nAMD. This may have contributed to the superior outcomes of PDT monotherapy observed among patients with PCV and CSC [19]. Notably, a reduction in the incidence of complications has been reported following combination treatment with anti-VEGF drugs and PDT [20,21]. Thus, current guidelines recommend using PDT in combination with anti-VEGF therapy.

### 2.3. Anti-VEGF + PDT Combination Therapy for the Management of Polypoidal Choroidal Vasculopathy

**Figure 4:**
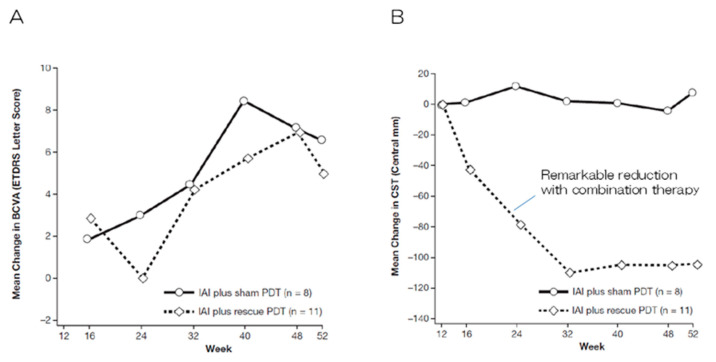
Sub-class analyses of the PLANET study [27]. (**A**) No differences were observed between the groups in terms of changes in the mean BCVA. (**B**) The combination of aflibercept and PDT remarkably reduced the mean central retinal thickness, whereas aflibercept monotherapy did not. This indicates the potent effect of PDT on improving anatomical outcomes regardless of vision.

Several clinical trials have been conducted to evaluate the outcomes of anti-VEGF + PDT combination therapy since the introduction of anti-VEGF therapy. Two randomized, double-blind prospective studies, the DENALI study and the MONT BLANC study conducted concurrently in North America [22] and Europe [23], respectively, compared the visual outcomes of a ranibizumab monotherapy group (administered monthly and as required after induction therapy in the DENALI study and MONT BLANC study, respectively) with those of a ranibizumab + PDT combination group (administered as needed after induction therapy in both studies; however, standard PDT was also compared with half-fluence PDT in the DENALI study). An improvement in mean visual acuity was observed after 12 months in the ranibizumab monotherapy and ranibizumab + PDT combination groups in both studies. No significant differences were observed between the two groups; however, greater improvements in visual acuity were observed in the ranibizumab monotherapy group. Typical nAMD accounts for the majority of cases in Europe and the US. The prevalence of PCV is low in these regions. Consequently, PDT, including combination therapy, has not been used in these regions since these studies. The EVEREST study [24], a randomized, double-blind, prospective comparative study involving 61 patients with PCV conducted in Asia at the same time as the abovementioned study, evaluated the outcomes of patients treated with ranibizumab alone, PDT alone, and a combination of ranibizumab + PDT. The regression rate of the polypoid lesions after six months, the primary endpoint, was 28.6%, 71.4%, and 77.8% in the ranibizumab alone, PDT alone, and ranibizumab + PDT combination groups, respectively, indicating significant improvement in all three groups in terms of the regression rates. No significant differences were observed among the three groups in terms of the changes in average visual acuity; nevertheless, the remarkable effect of combination therapy on PCV was particularly notable. Two approaches have been developed for the application of anti-VEGF + PDT combination therapy to PCV. The first approach involves initiating combination therapy from the beginning (or relatively early stages) to achieve a strong polyp regression effect of PDT for PCV. This approach is based on the findings of the EVEREST II trial [25], which reported a greater improvement in vision, the complete regression of polyp lesions, fewer injections, and non-inferiority in terms of safety among the patients with PCV in the ranibizumab + PDT group. The combination of ranibizumab and PDT yielded a significant improvement in terms of best-corrected visual acuity (BCVA) in patients with PCV at 12 months after commencing treatment in this study (average improvement of 5.1 letters and 8.3 letters on the ETDRS chart in the ranibizumab alone and ranibizumab + PDT groups, respectively; *p* = 0.013). In addition, a significant increase in the rate of the complete regression of polyp lesions (33.8% and 69.7% in the ranibizumab alone and ranibizumab + PDT groups, respectively; *p* < 0.001) and fewer injections (additional injections after initial induction therapy; on average 4.3 and 2.2 times in the ranibizumab alone and ranibizumab + PDT groups, respectively) were also observed. However, no differences were observed between the two groups in terms of safety. The EVEREST II study demonstrated the superiority of combination therapy in terms of all evaluated items, thereby renewing the interest in PDT. The 24-month results of the EVEREST II study also indicated the superiority of the ranibizumab + PDT group over the ranibizumab alone group [26]. The second approach for the application of anti-VEGF + PDT combination therapy to PCV involves using the expected add-on effect of PDT as a rescue treatment for PCV in cases wherein anti-VEGF therapy alone is ineffective. This approach is based on the findings of the PLANET study [27], which evaluated the effects of aflibercept. Aflibercept has more neutralizing targets than ranibizumab; moreover, it has a higher affinity for VEGF165. The PLANET study revealed that <6% of patients with PCV did not respond to initial treatment with aflibercept alone. No add-on effect of rescue PDT on visual improvement was observed in the group with a poor initial treatment response; consequently, combination therapy (i.e., PDT) was deemed unnecessary by some researchers. However, in clinical practice, the addition of PDT has been observed to result in rapid improvements in cases wherein aflibercept monotherapy failed to achieve the regression of polyp lesions or the disappearance of subretinal fluid [28]. The post hoc analysis of the PLANET study revealed a clear decrease in the central retinal thickness (which can be interpreted as the thickness of the lesion) of the PDT combination group compared with that of the sham PDT group (Figure 4). Therefore, combination therapy may be commenced in cases of PCV that fail to respond to initial anti-VEGF therapy. Aflibercept is the most commonly used anti-VEGF drug for nAMD in Japan at present. Consequently, the initial treatment of PCV is typically commenced with aflibercept alone. Nevertheless, combination therapy with PDT remains an important treatment option for cases exhibiting insufficient response or recurrence.

## 3. Current Conditions for Anti-VEGF + PDT Combination Therapy

**Figure 5:**
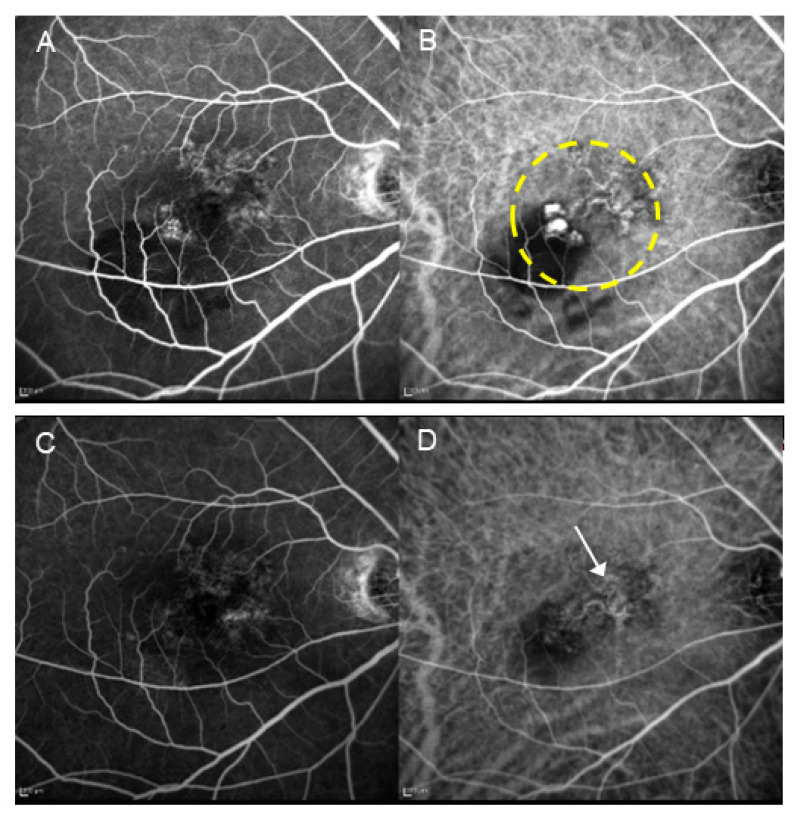
A patient with PCV (67-year-old male) who received combination therapy with aflibercept and PDT. (**A**) Pre-treatment FA shows suspected type 1 MNV. (**B**) Pre-treatment IA reveals the presence of blanching vascular networks and polypoid lesions. The area enclosed by the dashed line underwent laser irradiation. (**C**) Post-treatment FA shows faint tissue staining. (**D**) In IA after treatment, the regression of polypoid lesions is observed; however, the blanching vascular network remains (arrow). The occlusion of the choriocapillaris is minimal.

**Figure 6:**
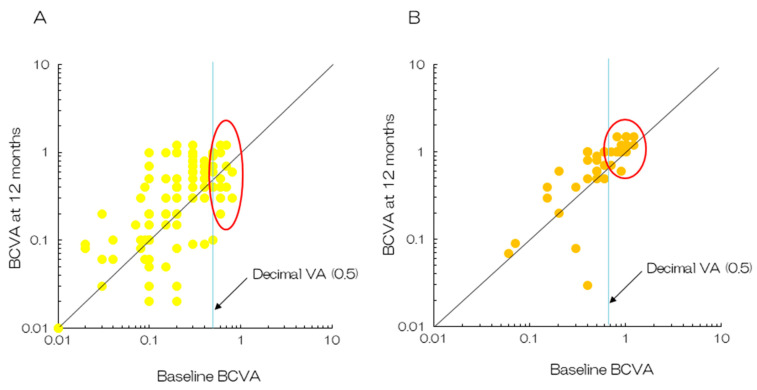
Plots of BCVA between baseline and 12 months after the initial treatment. (**A**) PDT monotherapy. Half of the patients with baseline decimal BCVA > 0.5 reported a decrease in their vision after 12 months (enclosed by the oval line) [11]. (**B**) The combination of aflibercept and PDT. Quite a few patients reported a decrease in vision after the initial treatment (enclosed by the oval line) [31].

A decrease in choroidal thickness is frequently observed in addition to the regression of the polyp lesions following PDT. Similar changes have been observed following combination therapy with anti-VEGF and PDT. However, compared with that observed following PDT alone, the atrophy of the choriocapillaris is observed less frequently following combination therapy (Figure 5) [29]. In addition, early complications, such as massive subretinal hemorrhage (which have been observed following PDT alone), are rarely observed following anti-VEGF + PDT combination therapy; the prevalence is almost identical to that observed following anti-VEGF therapy alone [28]. The criteria for the application of PDT have been set as follows based on the guidelines for PDT alone in Japan published in 2008 [4]: a maximum lesion diameter of ≤5400 µm and a pre-treatment decimal visual acuity of ≤0.5. However, the safety of anti-VEGF + PDT combination therapy has been gradually recognized in recent years; consequently, its application is expanding to cases with good visual acuity [30]. An analysis of the author’s own cases revealed that visual acuity declined 12 months after surgery in approximately half of the patients with PCV who had a pre-treatment decimal visual acuity of ≥0.5 subjected to PDT alone [11]. However, a decline in visual acuity was not observed in any of the patients who received aflibercept + PDT combination therapy (Figure 6) [31]. The author’s experience also indicates that aflibercept + PDT combination therapy is applicable to cases with a decimal visual acuity of ≤1.0. In addition, aflibercept + PDT combination therapy has exhibited a high safety profile, even for cases of PCV with large serous pigment epithelial detachments, which have a high risk of complications with PDT alone [32]. A previous report demonstrated no significant differences between the ranibizumab + PDT and ranibizumab monotherapy groups in terms of 5-year visual acuity, 5-year central retinal thickness, or incidence of macular atrophy [33]. PDT, through its combination with anti-VEGF therapy, can reduce vascular density in the choroid, reflecting the occlusion of the choriocapillaris and middle vessels in the choroid and stenosis of large vessels [34]. However, focusing on choroidal thickness, which was not considered in the guidelines at the time, revealed that the atrophic change observed in the macula after PDT was associated with a thin subfoveal choroidal thickness (226.2 ± 47.8 μm) [35]. Thus, a subfoveal choroidal thickness of >200 µm or <200 µm before treatment may be an indicator for the suitability of PDT. Another report mentioned that the pre-treatment choroidal thickness is related to visual acuity prognosis after combination therapy in patients with PCV [36].

## 4. Timing of PDT Combination

The timing of combination therapy varies depending on the purpose of using PDT. A prospective study (FUJISAN study) that randomly assigned patients with PCV to a group that received ranibizumab + PDT from the beginning or a group that received PDT later for cases that did not respond to ranibizumab alone revealed that significantly fewer total injections and fewer total PDTs were observed in the combination therapy group up to 12 months after commencing treatment [37]. This finding suggests that combination treatment with PDT during the early stages of PCV, when treatment sensitivity is high, is more effective. However, advancements in new anti-VEGF agents in the past 5 years have resulted in a decrease in the requirement for PDT in the treatment of nAMD, including PCV [38,39,40,41,42]. Consequently, PDT has mostly been performed as a rescue therapy if advanced anti-VEGF therapy fails to yield satisfactory outcomes in recent years. The favorable effects of rescue PDT on nAMD and PCV refractory to anti-VEGF therapy were demonstrated in a recent report [43,44]. An experimental study reported that pre-treatment with ranibizumab may enhance the angio-occlusive efficiency of PDT, thereby alleviating the endothelial inflammatory response, which gives it a great advantage over post-treatment with ranibizumab [45].

## 5. Standard vs. Reduced PDT

Several efforts, such as reducing the laser irradiation area, verteporfin dose, and laser dose, have been made to decrease the risks associated with PDT. The laser irradiation range in PDT was set to the greatest linear dimension (GLD) +1000 µm, corresponding to a margin of 500 µm around the area of the MNV lesion (originally including bleeding, fluorescent block, and pigment epithelial detachment), based on the FA and fundus images in the TAP and VIP studies [1,2]. This setting was set as the standard for PDT and used in the subsequent clinical practice of PDT. However, this setting often necessitates a large laser irradiation area, which is a risk factor for PDT-associated complications. Subsequently, indocyanine green angiography (IA)-guided PDT [46], which only targets MNV or PCV polyp lesions depicted by IA and minimizes the irradiation diameter, was proposed to reduce the risk of complications. However, lesions blocked by hemorrhages may be missed during treatment since subretinal hemorrhage sites are not included in the irradiation range in IA-guided PDT. Reduced-fluence PDT, which minimizes the amount of laser irradiation [47,48], and half-dose PDT, which halves the amount of administered verteporfin [49,50,51], have been used to avoid the complications of PDT. Similar effects of reduced-fluence PDT or half-dose PDT with standard PDT on visual and anatomical outcomes have been reported; however, half-dose PDT may yield a lower closure rate of polypoidal lesions than standard PDT in patients with PCV [49].

## 6. Long-Term Prognosis of Anti-VEGF + PDT Combination Therapy

The 6-year outcomes of patients with PCV recruited in the EVERESTII study were reported recently [52]. The monotherapy and combination treatment groups comprised 41 and 49 participants, respectively. No significant differences were observed between the monotherapy and combination groups in terms of the changes in BCVA from baseline to year 6 (−7.4 ± 23.0 and −6.1 ± 22.4 letters, respectively). However, the reduction in the central subfield thickness (CST) in the combination group was significantly greater than that in the monotherapy group at year 6 (−179.9 vs. −74.2 mm; *p* = 0.011). Fewer eyes in the combination group had subretinal fluid (SRF)/fluid/intraretinal fluid (SRF/IRF) compared with that in the monotherapy group at year 6 (35.4% vs. 57.5%; *p* = 0.032). The BCVA, CST, presence of SRF/IRF at 2 years, and number of anti-VEGF treatments during years 2–6 were identified as the factors associated with BCVA at year 6. The combination arm (odds ratio 0.45; *p* = 0.033), BCVA at year 2 (odds ratio 1.53; *p* = 0.046), and presence of SRF/IRF at year 2 (odds ratio 2.59; *p* = 0.042) were identified as the factors associated with the presence of SRF/IRF at year 6. Several reports evaluating the outcomes of ranibizumab + PDT combination therapy and aflibercept + PDT combination therapy in patients with PCV with up to 5 years of follow-up have been published [53,54,55,56]. The majority of these studies were retrospective observational studies; thus, variations have been observed between the reports in terms of the target case groups, initial treatment, and maintenance therapy. However, a meta-analysis revealed that the long-term (up to three years) prognosis of anti-VEGF + PDT combination therapy for PCV was superior to that of anti-VEGF therapy alone [57,58]. However, as maintenance therapy after initial treatment, which is important for evaluating the long-term course, the planned administration treat-and-extend (TAE) method, which is widely used for anti-VEGF monotherapy, is not common in anti-VEGF + PDT combination therapy, and the pro re nata (PRN) method is used in most cases. Therefore, it must be noted that the patients in the anti-VEGF therapy alone group, which was used for comparison, also received maintenance treatment using the PRN method. However, the evaluation of treatment effects should consider the economic burden of visual prognosis [59], given that the TAE method requires administering more injections than the PRN method.

## 7. PDT for the Management of PSD (Perspective of PDT)

**Figure 7:**
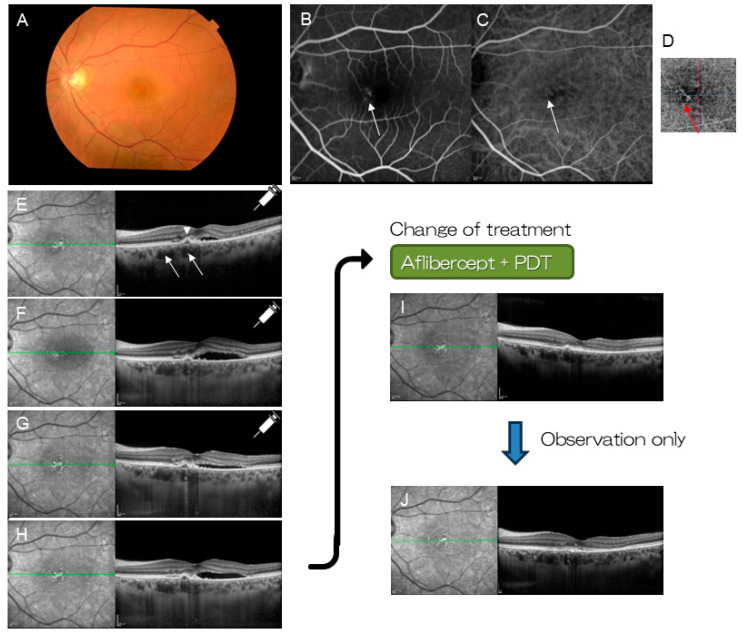
A patient with pachychoroid neovasculopathy (46-year-old female). (**A**) Color fundus photograph shows serous retinal detachment at the macula. Macular neovascularization (MNV) is suspected on FA (**B**) (arrow) and ICGA (**C**) (arrow). (**D**) Optical coherent tomography angiography (OCTA) visualizes MNV clearly (red arrow). (**E**) OCT shows pachyvessels (arrows) and small pigment epithelial detachment (arrowhead). Three monthly injections of aflibercept was selected as the initial treatment. (**F**) After the first injection. (**G**) After the second injection. (**H**) After the third injection. Subretinal fluid remained unchanged (or increased). Combination therapy with aflibercept and PDT was commenced. (**I**) Polypoidal lesion and subretinal fluid resolved within a month. (**J**) No recurrence occurred over 18 months of follow-up.

PSD, a group of macular diseases characterized by the presence of a thick choroid owing to the presence of large dilated choroidal vessels (pachyvessels), has remained a hot topic of research [60,61]. CSC, which is characterized by the presence of a thick choroid (and pachyvessels) and serous retinal detachment, is a representative subtype of PSD. PDT is highly effective in reducing choroidal thickness; consequently, reduced PDT, which minimizes the amount of verteporfin and laser irradiation compared with those associated with anti-VEGF therapy, has become the standard treatment for CSC globally. PDT has not been approved for the treatment of CSC in Japan and other countries at present, and the effects of off-label use of PDT have been reported in many cases. However, a randomized, double-masked, multicenter, placebo-controlled, Phase III clinical trial that aims to evaluate the efficacy and safety of reduced-fluence PDT for CSC is underway in Japan (Trial ID: jRCT2051230156). This study is expected to facilitate the national approval of PDT for the treatment of CSC. Pachychoroid neovasculopathy (PNV) accompanied by MNV in the pachychoroid is considered a type of nAMD according to the latest classification. PDT or combination therapy has been more effective than anti-VEGF therapy in many cases (Figure 7). Several recent studies have reported the favorable outcomes of combination therapy with PDT and anti-VEGF therapy in patients with PNV [30,62,63,64,65], which also responds to anti-VEGF monotherapy [66,67]. Various opinions regarding the treatment strategies for PNV have been voiced; hence, reaching a consensus will require additional time. The pathology of PSD must be detailed and the correct indications for PDT for this disease must be verified in the future.

## 8. Conclusions

PDT remains an effective intervention for specific subtypes of nAMD refractory to anti-VEGF therapy and several retinal and choroidal diseases (e.g., CSC, hemangioma, and other tumors) despite the recent advancements in anti-VEGF therapy for nAMD, including PCV and PNV. PDT may also reduce the number of anti-VEGF injections, which subsequently reduces patients’ burden over a long period. Thus, further applications of PDT should be considered in the future. Verteporfin and PDT have become unavailable in many countries owing to commercial reasons. Additional studies are required to develop new photosensitizers (more specifically incorporated into MNV tissue) or laser conditions (wavelength, power, irradiation time) to improve the risk/benefit ratio. However, PDT remains an essential treatment modality in present and future ophthalmology.

## Data Availability

No new data were created in this study.
